# A novel strategy for comprehensive optimization of structural and operational parameters in a supersonic separator using computational fluid dynamics modeling

**DOI:** 10.1038/s41598-021-01303-5

**Published:** 2021-11-08

**Authors:** Sina Nabati Shoghl, Abbas Naderifar, Fatola Farhadi, Gholamreza Pazuki

**Affiliations:** 1 Department of Chemical Engineering, Amirkabir University of Technology (Tehran Polytechnic), Tehran, Iran; 2 Department of Chemical and Petroleum Engineering, Sharif University of Technology, Azadi Ave., Tehran, Iran

**Keywords:** Chemical engineering, Mechanical engineering

## Abstract

In this study, the effects of several structural and operational parameters affecting the separation efficiency of supersonic separators were investigated by numerical methods. Different turbulence models were used and their accuracies were evaluated. Based on the error analysis, the V2-f turbulence model was more accurate for describing the high swirling turbulent flow than other investigated turbulence models. Therefore, the V2-f turbulence model and particle tracing model were selected to optimize the structure of the convergence part, the diffuser, the drainage port, and the swirler. The cooling performance of three line-type in the convergent section were calculated. The simulation results demonstrated that the convergent section designed by the Witoszynski curve had higher cooling depth compared to the Bi-cubic and Quintic curves. Furthermore, the expansion angle of 2° resulted in the highest stability of fluid flow and therefore was selected in the design of the diffuser. The effect of incorporating the swirler and its structure on the separation performance of supersonic separator was also studied. Three different swirler types, including axial, wall-mounted, and helical, were investigated. It was observed that installing the swirler significantly improved the separation efficiency of the supersonic separator. In addition, the simulation results demonstrated that the separation efficiency was higher for the axial swirler compared to the wall-mounted and helical swirlers. Therefore, for the improved nozzle, the swirling flow was generated by the axial swirler. The optimized axial swirler was constructed from 12 arced vanes each of which had a swirl angle of 40°. For the optimized structure, the effects of operating parameters such as inlet temperature, pressure recovery ratio, density, and droplet size was also investigated. It was concluded that increasing the droplet size and density significantly improved the separation efficiency of the supersonic separator. For hydrocarbon droplets, the separation efficiency improved from 4.6 to 76.7% upon increasing the droplet size from 0.1 to 2 µm.

## Introduction

Natural gas is generally saturated with heavy hydrocarbons (HCs) and water vapor when extracted from the reservoir^1^. The presence of water vapor in the natural gas pipelines decreased the heating value and transportation capacity and increased the operational cost. Dew point depression of natural gas is crucial from the industrial and economic point of view. Conventional methods have been used for natural gas dehydration and natural gas liquid (NGL) recovery, such as adsorption, absorption, membrane separation, and cryogenic processes. Among conventional methods, the cryogenic processes are the only separation technique that can depress both water and HC dew point simultaneously. The supersonic separator (3S) is a novel separation equipment that integrates phase change and separation technique in a single compact device. Furthermore, due to the very high gas velocity and short residence time, the gas hydrate cannot be formed inside the 3S, and the need for chemical inhibitor injection is eliminated. Generally, chemical hydrate inhibitor like Tri ethylene glycol causes pollution and environmental problems. NGL recovery, water, and HC dew point depression, natural gas sweetening, natural gas dehydration, and liquefied petroleum gas extraction are 3S’s applications in the natural gas industry^2^. Compared with traditional separation techniques, like the Joule–Thomson process and turbo-expander, at the same operational condition, the 3S can achieve lower temperature and higher NGL recovery^3^. The first engineering group^4^ installed its Twister® in PETRONAS offshore plant^5^. The capacity of this dehydration system was about 600 MMSCFD, and each system contained six twister nozzles. Several field tests were performed in the SINOPEC Shengli oilfield^6^ to characterize the real influence of this equipment on dehydrating. Their results demonstrated that this equipment not only depress the natural gas dew point but also it separates the light HCs.

The performance of the 3S highly depends on the structure of the nozzle. To improve the cooling performance and rate of liquefaction of natural gas condensable, the nozzle structure should be optimized. Several works investigated the effect of 3S structure. For instance, Mahmoodzadeh and Shahsavand^7^ optimized the converging–diverging nozzle structure by the generalized radial basis function artificial neural networks. Almeida^8^ observed that divergent angle, throat length, and diffuser shape have significant influences on the nozzle performance. Wen et al.^9^ selected three types of diffusers, including the curved wall, second throat diffuser and conical structure to investigate the gas flow behavior. They observed that the conical diffuser with high-pressure recovery was more suitable for the 3S. Cao et al.^10^ investigated the role of an ellipsoidal core experimentally and by the computational fluid dynamics (CFD) modeling. They observed that installing this core decreased the flow resistance. Qingfen et al.^11^ reported that installing the inner body (central core) improved the separation efficiency and decreased the energy loss. Yang et al.^12^ observed that the separation efficiency was improved by installing an inner body. They attributed this phenomenon to the enhancement of the centrifugal force. Park et al.^13^ reported that the nozzle performance deteriorated by increasing the divergent angle. Wen et al.^14^ designed a new convergent-divergent nozzle and investigated the influence of nozzle structure on the separation performance by numerical study. Wen et al.^15^ used the CFD modeling to investigate the influence of blade angles on the gas swirling behavior. Majidi and Farhadi^16^ investigated the influence of swirler on the shockwave position. They observed that an increase in the blade number, height, and end angle resulted in the shockwave position shifted toward the nozzle outlet.

The influence of several operational parameters, including inlet temperature, droplet size, inlet pressure, mass flow rate, and shock wave position, have been studied in previously published works^17–19^. Hengwei et al.^20^ experimentally investigated the natural gas dehydration using the 3S. They studied the influence of various parameters, including pressure loss ratio, inlet temperature, and gas flow rate, on the dehydration performance. Besides, they observed a dew point depression of about 20 °C at the optimal condition. A wet stream model was also developed^21^ to evaluate the flow structure through the 3S considering simultaneous shock wave occurrence and non-equilibrium condensation. To investigate the performance of 3S the influence of the inlet saturation and inlet sub-cooling on the condensation behavior was investigated. Karimi and Abdi^17^ employed the MATLAB® and HYSYS® software to predict the influence of the operational parameters on gas flow through the Laval nozzle. A 2D model for characterization of swirling flow inside the converging–diverging nozzle was developed by Malyshkina et al.^18,22^. They reported that the separation efficiency depended on the inlet pressure, inlet temperature, inlet velocity and the composition of working fluid. The potential of clean natural gas dehydration by non-equilibrium condensations in a 3S was also investigated^23^. It was observed that decreasing inlet temperatures provided earlier condensing onset and improved gas processing capacity through the 3S. Liu et al.^24^ employed the discrete particle model (DPM) to predict the particle flow in a converging–diverging nozzle. They assumed that the particle diameter was about 10–50 µm which was higher than the usual particle size (0.1–2 µm)^25^. Secchi et al.^26^ defined two typical sizes, including 0.6 µm and 4 µm for the liquid droplets. One of the main advantages of this converging–diverging nozzle is the selective separation of the desired component by adjusting the PRR. Yang et al.^27^ reported that shock wave position was more affected by pressure boundary conditions rather to temperature boundary conditions. The effect of inlet temperature, inlet pressure, and outlet pressure on the location of shockwave were investigated by Karimi and Abdi^17^. They reported that by decreasing the inlet temperature and increasing the outlet pressure, the shockwave position moves earlier.

With the development of computing capacity, numerical modeling plays a crucial role in describing gas flow behavior through separators. Several numerical studies have been conducted on the 3S. For example, Bao et al.^28^ developed a steady-state mathematical model for phase equilibrium calculation through the 3S. They employed Benedict-Webb-Rubin-Starling (BWRS) equation of state (EoS) and compared their model output with field test data. CFD modeling played a crucial role in the development and optimization of the 3S. To describe the CO_2_ condensing flow through the 3S, a CFD model was developed by Wen et al.^29^ where it predicted accurately the distribution of the static temperature. Haghighi et al.^30^ reported that the CFD modeling is a suitable method for analyzing the 3S. Vaziri et al.^31^ employed the CFD modeling to optimize the angular, axial, and radial components of velocity. The DPM was used by Yang and Wen^12^ to investigate the droplet motion inside a 3S equipped with a delta wing. Yang et al.^32^ reported that the influence of steam condensation on the performance of 3S ejector was not understood comprehensively due to the dry gas assumption. To understand the intricate feature of the steam condensation a wet steam model based on the CFD was developed by them. Liu et al.^24^ investigated the flow behavior and separation performance of a 3S by the Reynolds stress model (RSM) and DPM and compared it with recorded industrial data of the wet gas.

The behavior of supersonic flow through the 3S was not understood completely due to complicated interaction of swirling flow with shock wave occurrence and phase change^21^. The aim of this study is to evaluate the structural and operational parameters that affect the separation efficiency of a 3S by the particle tracing model. In this work, in addition to studying the factors affecting the separation efficiency of 3S, a novel method for optimization of 3S structure was presented. Generally, any modification that improves the collection efficiency of 3S reduces its cooling performance. As a result, it is necessary to find structural and operational conditions in which these two parameters have optimal values. Most of the previously published work has focused on optimizing one of the parameters studied, including cooling performance and collection efficiency parameters. Therefore, a blending technique was used in which the separation efficiency was defined as a function of the cooling performance and collection efficiency. In this technique, the separation efficiency, which is defined as the product of the cooling performance and collection efficiency, was selected as the objective function. Once this technique was employed, the structures of the nozzle, the drainage port, and the swirler were optimized. To the best of the authors’s knowledge, there is a lack of complete optimization of the structural and operational parameters in a 3S using the developed method.

## Mathematical modeling

The modeling of gas flow through the 3S is very complicated due to the swirling flow, supersonic velocity, and shockwave formation. Therefore, the CFD modeling was employed to attain a clear view of the natural gas flow behavior inside the supersonic swirling separator. The 3S operation is based on the three main principles: gas expansion, cyclonic separation, and pressure recovery at the diffuser. This study contains two sections: first, analyzing the influence of structural parameters and second, analyzing the influence of operational parameters on the separation performance. The mass, momentum and energy equations were simultaneously solved to describe the compressible flow behavior through the converging–diverging nozzle. The momentum conservation equation is as follow:

1 $$ \rho {{Dv} \mathord{\left/ {\vphantom {{Dv} {Dt}}} \right. \kern-\nulldelimiterspace} {Dt}} = - \nabla p + \rho \vec{g} - \nabla .\tilde{\tau } $$

The energy conservation equation is as follow:

2 $$ \rho C_{p} {{DT} \mathord{\left/ {\vphantom {{DT} {Dt = k\nabla^{2} T}}} \right. \kern-\nulldelimiterspace} {Dt = k\nabla^{2} T}} + Q + Q_{p} + Q_{vd} $$

The continuity equation is as follow:

3 $$ {{\partial \rho } \mathord{\left/ {\vphantom {{\partial \rho } {\partial t}}} \right. \kern-\nulldelimiterspace} {\partial t}} + \nabla .\left( {\rho \vec{v}} \right) = 0 $$

where *P*, *k*, *µ*,* T*,* ρ*, *t*, $\vec{v}$, *Q*_*vd*_, *Q*_*p*_, *C*_*p*_ and *Q* are pressure, thermal conductivity, viscosity, temperature, density, time, velocity vector, viscous dissipation, pressure work, heat capacity at constant pressure, and heat source, respectively. The stress tensor ($\tilde{\tau }$) was determined by Eq. (4)

4 $$ \tilde{\tau } = \mu \left[ {\left( {\nabla .\vec{v} + \vec{v}^{T} } \right) - {\raise0.7ex\hbox{$2$} \!\mathord{\left/ {\vphantom {2 3}}\right.\kern-\nulldelimiterspace} \!\lower0.7ex\hbox{$3$}}\nabla .\vec{v}I} \right] $$

**Figure 1:**
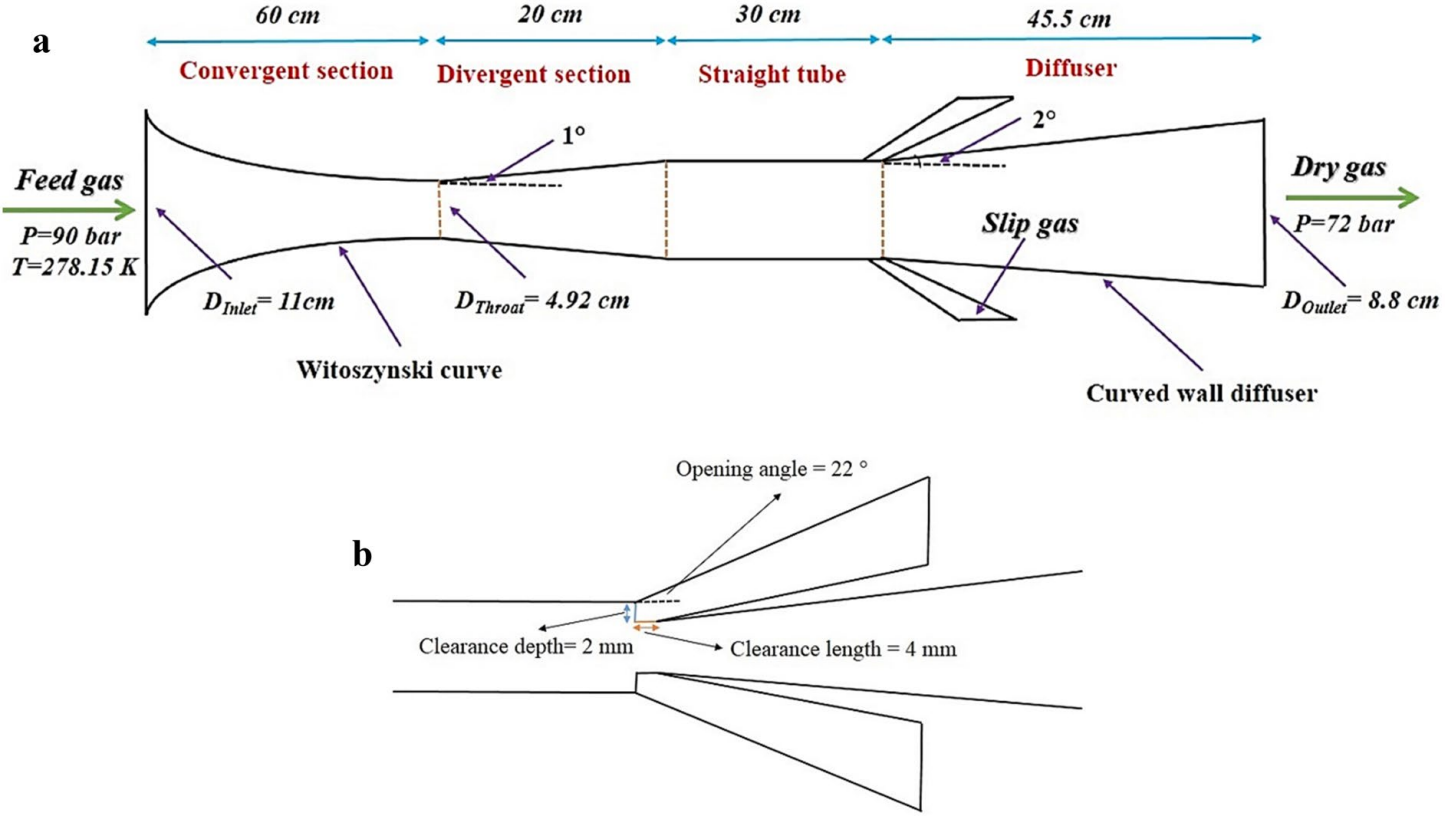
The 2D schematic of (**a**) converging–diverging nozzle, (**b**) drainage port.

The total length of the nozzle was 155.5 mm, involving the convergent section of 60 mm and the divergent section of 20 mm. The opening angle of the drainage port was 22°^33^. In this study, the slope and line-type of the central body were the same as the nozzle wall in the convergent and divergent sections. Other defined dimensions of the considered nozzle is presented in Fig. 1. The pressure boundary condition was assigned for the inlet and outlet of the nozzle. The outlet pressure was adjusted at 80% of inlet pressure. In addition, no-slip along with adiabatic boundary conditions were defined for the wall of the separator.

The throat diameter should be specified to attain sonic velocity at the throat with the highest pressure recovery ratio (PRR). One of the most important parameters for characterization of the separator performance is the PRR, which is defined as follow:

5 $$ PRR = \frac{{P_{out} }}{{P_{in} }} \times 100\% $$

where *P*_*out*_ and *P*_*in*_ are the outlet and inlet pressure of the nozzle, respectively. In this study, the 3S was employed to recover the heavier HCs from a natural gas stream. The volumetric flow rate of this natural gas stream was about 4 MMSCMD. A specified natural gas mixture from an NGL recovery unit, with composition shown in Table 1, was considered for optimization of separator geometry.

**Table 1:** The natural gas composition in mole fraction.

Component (mol%)	CH_4_	C_2_H_6_	C_3_H_8_	i-C_4_H_12_	n-C_4_H_12_	i-C_5_H_12_	n-C_5_H_12_	C_6_H_14_	C_7_+	CO_2_	N_2_	H_2_O
Composition	89.4	3.7	1.55	0.31	0.42	0.1	0.07	0.04	0.01	1.1	3.3	0.03

### Particle tracing model and induced drag force

When the natural gas temperature is below the saturation temperature, the phase change initiates; therefore the gas phase partially condenses into the liquid phase. This multi-phase flow moved into the divergent section, and liquid droplets are separated due to the centrifugal force and density difference between the dispersed and continuous phase. The liquid droplets move circularly through the 3S under the combined influence of drag forces, gravity forces, and centrifugal forces. To track the behavior of liquid droplets through this converging–diverging nozzle, the particle tracing model was used, which is based on the newton’s second law of motion. This model was used to determine the liquid droplets behavior and the separation efficiency. This 3D CFD modeling was developed by the following assumptions:

- The gravity force is neglected due to its negligible influence in centrifugal separation.
- The size and shape of the liquid droplets are assumed to remain constant and spherical.
- The mutual interaction between droplets is neglected.

The droplets behavior through the nozzle was described by force balance on a droplet as follows:

6 $$ F = \frac{{d\left( {mv} \right)}}{dt} $$

where *m* and *v* are the mass and volume of droplets, respectively. The drag force (*F*_*D*_) was estimated by Schiller–Neuman^34^ equation as follow:

7 $$ F_{D} = \frac{{3\mu C_{D} {\text{Re}}_{r} }}{{4\rho_{p} d_{p}^{2} }}mp\left( {u^{\prime} - v} \right) $$

where $u^{\prime}$ was calculated by Eq. (8):

8 $$ u^{\prime} = u + \Delta u \, \Delta u = \xi \sqrt{\frac{2k}{3}}  $$

where *ξ* shows the un-correlated Gaussian number vector with unit variance. The drag coefficient was determined by Eq. (9)^34^:

9 $$ C_{D} = \frac{24}{{{\text{Re}}_{r} }}\left( {1 + 0.15{\text{Re}}_{r}^{0.687} } \right) $$

where *Re*_*r*_ was calculated as follow:

10 $$ {\text{Re}}_{r} = \frac{{\rho \left| {u - v} \right|d_{p} }}{\mu } $$

In this study, two kinds of liquid droplets, including water and HC droplets (natural gas condensate), were considered. The collection efficiency of liquid droplets was determined by the following equation:

11 $$ \eta = \frac{{n_{sep.} }}{{n_{sep.} + n_{escape} }} \times 100\% $$

where *n*_*sep*._ and *n*_*escape*_ are the number of separated and escaped liquid droplets, respectively. The collection efficiency showed the capability of each structure to separate the liquid droplet from the gas phase. The liquid droplets were released at the nozzle inlet, and the separated droplets counted at the drainage port. In this study, the density of liquid droplets was defined about 998 kg/m^3^ and 584 kg/m^3^ for water and natural gas condensate, respectively.

### Nozzle line type and swirler structure

The 3S is composed from three main sections including:

- *Laval nozzle* as the most common type of Venturi nozzle, the natural gas temperature and pressure decrease, and its velocity increases in this region, and phase change may occur in this part of the nozzle.
- *Swirler* this equipment generates a great swirling flow to separate liquid droplets from the gas phase.
- *Diffuser* in this part, the pressure of the working fluid is recovered, and its velocity decreased significantly.

The curvature of convergent and diffuser sections, significantly influenced the separator cooling performance. The considered line-type for the convergent section and diffuser were depicted in Fig. S.1. The convergent section of the Laval nozzle was designed by the cubic polynomials presented in Table 2. The role of the Laval nozzle is providing very low temperature and pressure for liquefaction of water vapor and heavy HCs, while the role of swirler is generating a strong swirl flow for separation of liquid droplets. Therefore, the cooling performance and the rate of liquid droplet formation can be improved by optimizing the structure of the Laval nozzle. While, after the formation of these droplets, a robust centrifugal force should be generated by an improved swirler to separate the liquid droplets. The Laval nozzle and the swirler should be designed appropriately to prevent disturbance and maintain the flow stability downstream of the swirler. The diffuser geometry can also be improved by variation of expanding angle and using a curved or linear wall for this section. The role of the diffuser is converting kinetic energy to pressure energy. Another modification that improves the collection efficiency and cooling performance is inserting a channel with a constant cross-sectional area after the Laval nozzle. To examine the separation behavior, a 3D CFD model was developed, and the influence of various modifications on the separation performance at different operating conditions was studied.

**Table 2:** The cubic polynomials used for designing the convergent section of nozzle^2^.

$\frac{{r - r_{Th} }}{{r_{In} - r_{Th} }} = 1 - \frac{1}{{X_{m}^{2} }}\left( \frac{x}{L} \right)^{3} \, \left( {\frac{X}{L} \le X_{m} } \right)$	Bi-cubic curve	(12)
$$\frac{{r - r_{Th} }}{{r_{In} - r_{Th} }} = 1 - \frac{1}{{\left( {1 - X_{m} } \right)^{2} }}\left( {1 - \frac{x}{L}} \right)^{3} \, \left( {\frac{x}{L} > X_{m} } \right)$$		
$r = \frac{{r_{Th} }}{{\sqrt {1 - \left[ {1 - \left( {{{r_{Th} } \mathord{\left/ {\vphantom {{r_{Th} } {r_{In} }}} \right. \kern-\nulldelimiterspace} {r_{In} }}} \right)^{2} } \right]\frac{{\left( {1 - \left( \frac{x}{L} \right)^{2} } \right)^{2} }}{{\left( {1 + \frac{{x^{2} }}{{3 \times L^{2} }}} \right)^{3} }}} }}$	Witoszynski curve	(13)
$\frac{{r - r_{Th} }}{{r_{In} - r_{Th} }} = 1 - 10\left( \frac{x}{L} \right)^{3} + 15\left( \frac{x}{L} \right)^{4} - 6\left( \frac{x}{L} \right)^{5}$	Quintic curve	(14)

**Figure 2:**
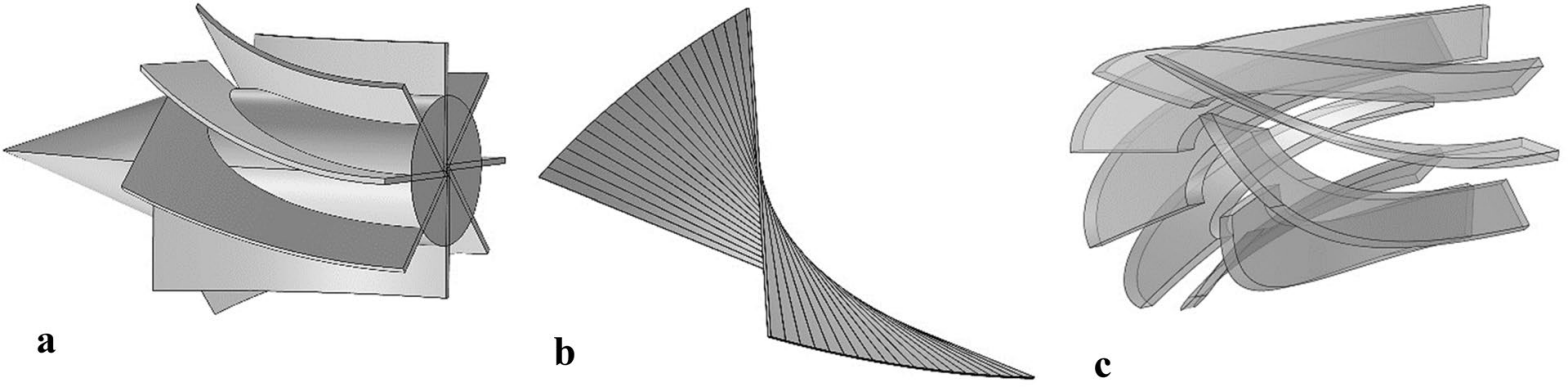
The structure of different studied swirler (**a**) axial swirler with a cone core, (**b**) helical swirler, (**c**) wall-mounted swirler.

**Figure 3:**
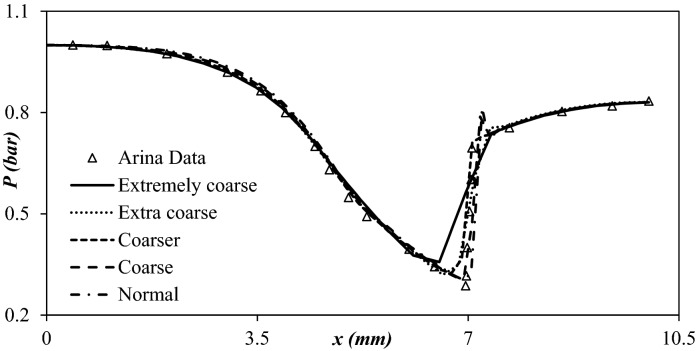
Comparison of simulation results with Arina’s data in different mesh sizes.

The above analytical formula gave the contours of the convergent part, where *r*, *r*_*Th*_, *r*_*In*_ and *L* are the convergent radius at distance *x* from the inlet, the throat radius, the inlet radius, and the convergent length, respectively. The divergent section of the Laval was determined using the divergent angle (*α*) and throat radius (*r*_*Th*_) as follow:

15 $$ Tan\left( \alpha \right) = \frac{{r - r_{Th} }}{{x - x_{Th} }} $$

where *α*, *x*_*th*_ and *r*_*th*_ are the divergent angle, axial coordinate, and radius of throat, respectively. The *α* = *1°* was assumed for all studied structures in this paper. Additional length can raise the residence time and separation performance. Therefore, a constant cross-sectional area channel was inserted after the divergent section of the Laval nozzle. Diffuser plays a crucial role in the conversion of kinetic energy to pressure energy. The radius of each cross-sectional area of curved wall diffuser was calculated by the Eq. (16):

16 $$ r = \frac{{r_{1} }}{{\sqrt {1 + \left( {\frac{{r_{1}^{2} }}{{r{}_{2}^{2} }} - 1} \right)\frac{x}{L}} }} $$

where *r*_*1*_ and *r*_*2*_ are the inlet and outlet radius of the diffuser, and *L* shows the diffuser’s length. To improve the collection efficiency of water vapor and heavy HC in the 3S, various structures, including wall-mounted cyclone, helical swirler, and axial swirler, were designed (Fig. 2). Then, the natural gas flow characteristics inside the 3S equipped with these swirler, were investigated numerically. In this study, the static vanes line were designed with one arc and by Eq. (17)^12^:

17 $$ y = \sqrt {\left( {{\raise0.7ex\hbox{${0.5}$} \!\mathord{\left/ {\vphantom {{0.5} {\sin \left( {{\theta \mathord{\left/ {\vphantom {\theta 2}} \right. \kern-\nulldelimiterspace} 2}} \right)}}}\right.\kern-\nulldelimiterspace} \!\lower0.7ex\hbox{${\sin \left( {{\theta \mathord{\left/ {\vphantom {\theta 2}} \right. \kern-\nulldelimiterspace} 2}} \right)}$}}} \right)^{2} - \left( {x - 0.5} \right)^{2} } - \frac{0.5}{{\tan \left( {{\theta \mathord{\left/ {\vphantom {\theta 2}} \right. \kern-\nulldelimiterspace} 2}} \right)}} $$

where θ is the vane angle. Two locations for installing the swirler were suggested: one at the separator entrance (for axial swirler), and the other at the constant cross-sectional area channel (for wall mounted cyclone and helical swirler) before the drainage port. The axial swirler was composed of a core and several static vanes (Fig. 3a). The helical swirler (Fig. 2b) was installed in the straight tube, formed from several parallel blades. The front end of the helical and wall-mounted swirler (Fig. 2c) are placed at x = 0.8 m, and their rear end is at the x = 0.9 m. For all swirler, a fixed length of 10 cm was considered. A distance should be considered between the helical and wall-mounted swirler with the drainage port because the central droplets are far away from the separator wall. Therefore, this distance is required to provide enough time for reaching the droplets toward the separator wall. In this study, the configuration of the swirler was optimized by numerical analysis. Another important factor in nozzle behavior was the drainage port structure. The drainage system was employed to remove the separated droplets from the natural gas stream. Two types of drainage structures, including flush type (clearance depth = 0 mm) and internal extension drainage (clearance depth˃0 mm), were considered, and their structures were optimized.

The cooling performance and the collection efficiency are two main factors that influence the nozzle separation performance. Generally, the balance between the cooling performance and collection efficiency should be considered to achieve a nozzle with proper behavior. This study focuses on these two factors for improving the nozzle performance. The ability of dew point depression by the 3S depends on the lowest temperature inside the nozzle. The condensation increases with cooling performance, an indication of the nozzle separation efficiency. Therefore, the cooling performance is an important parameter for the characterization of separator performance. The cooling performance and separation efficiency were defined by Eqs. (18) and (19), respectively:

18 $$ \zeta = \frac{{T_{in} - T_{\min } }}{{T_{in} - T_{\min - Simple} }} \times 100\% $$

19 $$ \gamma = \eta \times \zeta $$

where *ζ*, *T*_*min-simple*_, *T*_*in*_, *T*_*min*_, and γ are the cooling performance, the minimum temperature inside the optimized nozzle (without swirler), inlet temperature, the minimum temperature inside the modified nozzle, and separation efficiency, respectively.

### Mesh independency

The mesh quality and density have a crucial role in the accuracy of the model output. The grid independence test was conducted to achieve the optimum mesh size. In order to attain mesh independency, the considered domain was meshed by the extremely coarse (40,316 cells), extra coarse (71,869 cells), coarser (116,792 cells), coarse (207,927 cells), and normal grids (370,543 cells), respectively. A tetrahedral mesh was employed for the 3S. Mesh was generated for each section by COMSOL Multiphysics® Version 5.4” software. In this section, the pressure profile along the nozzle length was selected to achieve mesh independency. The convergence criterion of mass, momentum, and energy equations are 10^–3^. The time-dependent developed equations were solved using PARDISO solver. Furthermore, both direct multi-grid and iterative solvers were used in this paper. The Arina’s data^35^ were used for the mesh independency test, with dimensions:

20 $$ \begin{gathered} A\left( x \right) = 2.5 + 3\left( {{\raise0.7ex\hbox{$x$} \!\mathord{\left/ {\vphantom {x {x_{th} }}}\right.\kern-\nulldelimiterspace} \!\lower0.7ex\hbox{${x_{th} }$}} - 1.5} \right)\left( {{\raise0.7ex\hbox{$x$} \!\mathord{\left/ {\vphantom {x {x_{th} }}}\right.\kern-\nulldelimiterspace} \!\lower0.7ex\hbox{${x_{th} }$}}} \right)^{2} \, x \le x_{th} \hfill \\ A\left( x \right) = 3.5 - {\raise0.7ex\hbox{$x$} \!\mathord{\left/ {\vphantom {x {x_{th} }}}\right.\kern-\nulldelimiterspace} \!\lower0.7ex\hbox{${x_{th} }$}}\left( {6 - 4.5\left( {{\raise0.7ex\hbox{$x$} \!\mathord{\left/ {\vphantom {x {x_{th} }}}\right.\kern-\nulldelimiterspace} \!\lower0.7ex\hbox{${x_{th} }$}}} \right) + \left( {{\raise0.7ex\hbox{$x$} \!\mathord{\left/ {\vphantom {x {x_{th} }}}\right.\kern-\nulldelimiterspace} \!\lower0.7ex\hbox{${x_{th} }$}}} \right)^{2} } \right) \, x \ge x_{th} \hfill \\ \end{gathered} $$

where *x*_*th*_ and *A*_*th*_ are equal to 50 mm and 100 mm^2^, respectively. Air is the working fluid, and inlet temperature and pressure are equal to 288 K and 100,000 Pa, respectively. In addition, the outlet pressure is 83,049 Pa. Figure 3. provides the model prediction for pressure distribution of air in the 3S for the Arina’s nozzle^35^. It can be observed that the prediction of the developed model was in complete agreement with Arina’s data for the entire length of the separator. Furthermore, the coarse mesh size provided appropriate grid independency for the considered separator. While coarse mesh configuration showed grid independency, but, for exact prediction of shockwave position, the simulation was conducted by normal mesh size.

Furthermore, The Grid Convergence Index (GCI) for the finest mesh size was determined by the Eq. (21)^36,37^:

21 $$ GCI_{21} = \frac{{F_{s} \left| \varepsilon \right|}}{{r_{21}^{p} - 1}} $$

The calculated GCI (for normal grid size) for all considered data on the Ariana’s pressure profile was as follows:

22 $$ \frac{{GCI_{32} }}{{r^{p} GCI_{21} }} \approx 1 $$

Therefore, this conclusion certified that the normal mesh size was mesh independent.

### Equation of state (EoS)

The considered operation were conducted at very low temperature and very high pressure where the natural gas state is far from a perfect gas behavior. Therefore, the appropriate EoS should be used to describe the natural gas properties. The prediction of Joule–Thomson inversion curve (JTIC) is crucial not only for cryogenic processes but also for testing the EoS ability. The location of JTIC can be predicted^38^ from solving Eq. (23) along with any EoS, simultaneously:

23 $$ T\left( {{\raise0.7ex\hbox{${\partial p}$} \!\mathord{\left/ {\vphantom {{\partial p} {\partial T}}}\right.\kern-\nulldelimiterspace} \!\lower0.7ex\hbox{${\partial T}$}}} \right)_{V} + V\left( {{\raise0.7ex\hbox{${\partial p}$} \!\mathord{\left/ {\vphantom {{\partial p} {\partial V}}}\right.\kern-\nulldelimiterspace} \!\lower0.7ex\hbox{${\partial V}$}}} \right)_{T} = 0 $$

where *T, P,* and *V* are the temperature, pressure, and molar volume, respectively. As shown in Table 1, methane was the main component of considered natural gas. The JTIC was plotted for this component and compared with the experimental data in published work by the authors^38–41^. Results demonstrated that the low-temperature region is predicted accurately by considered EoS, while at the high-temperature branch, only Soave–Redlich–Kwong (SRK) EoS predicts this curve appropriately. In conclusion, among Van der Waals, Peng-Robinson, and SRK EoS, the SRK EoS was selected to estimate the thermodynamic properties of the studied gas.

The sound speed can be estimated by Eq. (24)^14^:

24 $$ C = \sqrt {\frac{\lambda P}{\rho }} $$

where *ρ*, *P* and *λ* are the natural gas density, pressure, and ratio of specific heat capacity (C_p_/C_v_), respectively. The Mach number can be written as^42^:

25 $$ Ma = \frac{{\vec{v}}}{{\sqrt {\frac{\gamma P}{{\rho_{G} }}} }} $$

The sound speed for SRK EoS can be determined by Eq. (26):

26 $$ C^{2} = - \frac{{\lambda v^{2} }}{M}P_{J} $$

where *M* and *C* are the molecular weight and sound speed, respectively. The *λ* and *P*_*J*_ are determined by the following equations:

27 $$ \lambda = {\raise0.7ex\hbox{${C_{P} }$} \!\mathord{\left/ {\vphantom {{C_{P} } {C_{V} }}}\right.\kern-\nulldelimiterspace} \!\lower0.7ex\hbox{${C_{V} }$}} $$

28 $$ P_{J} = \left( {\frac{\partial P}{{\partial V}}} \right)_{T} = - \frac{RT}{{\left( {V - b} \right)^{2} }} + \frac{{\left( {2V + b} \right)\left( {a\alpha } \right)}}{{V^{2} \left( {V + b} \right)^{2} }} $$

### Turbulence model

**Figure 4:**
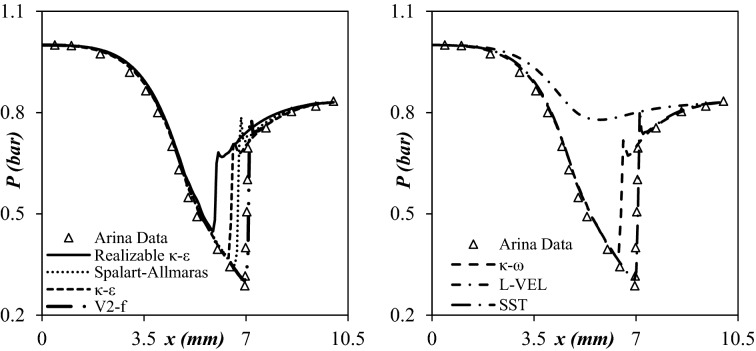
Predicted pressure profiles by different turbulence models.

Due to the strong swirling flow inside the 3S, the appropriate turbulence model should be employed to describe natural gas behavior. The most common turbulence models for describing the swirling flow inside the 3S contain κ-ε, κ-ω, realizable κ-ε, shear stress transport (SST), and RSM^3,43,44^. Therefore, in this study, seven common turbulence models, including κ-ε, κ-ω, V2-f, SST, L-VEL, Realizable κ-ε, and Spalart–Allmaras, were investigated and analyzed. The predicted pressure profile through all the seven turbulence models was shown in Fig. 4. In addition, the calculated average absolute relative deviation percent (AARD%) for studied turbulence models were summarized in Table 3. Figure 4 demonstrates that The V2-f turbulence model was more accurate for describing the high swirling turbulent flow than other investigated turbulence models. The error analysis (Table 3) also certified this conclusion. Therefore, the V2-f turbulence model was employed to model complex turbulent flow inside the nozzle due to its effective results. Also, the V2-f turbulence model was considered for problems with the strong swirling flow like hydro-cyclone in “COMSOL Multiphysics® Version 5.4” software^45^. Therefore, the V2-f turbulence model was used to predict the swirling flow behavior inside the nozzle. This model used a new parameter called the elliptic blending function (α) for describing the natural gas flow. Moreover, an automatic wall treatment was considered in this turbulence model, which switches between a low-Reynolds-number formulation and a wall function formulation. This property gives a robust formulation to this turbulence model (V2-f).

**Table 3:** AARD% of different turbulence models (Arina data).

Turbulence model	AARD%	Turbulence model	AARD%
κ-ε	25.39	Spalart–Allmaras	21.30
SST	16.71	κ-ω	20.56
V2-f	8.65	L-VEL	43.47
κ-ε realizable	30.28		

In this paper, the V2-f turbulence model was used to characterize the swirling flow behavior inside the nozzle, as follow:

29 $$ \rho \left( {u.\nabla } \right)k = \nabla .\left[ {\left( {\mu + \frac{{\mu_{T} }}{{\sigma_{K} }}} \right)\nabla k} \right] + P_{k} - \rho \varepsilon $$

30 $$ \rho \left( {u.\nabla } \right)\varepsilon = \nabla .\left[ {\left( {\mu + \frac{{\mu_{T} }}{{\sigma_{\varepsilon } }}} \right)\nabla \varepsilon } \right] + \frac{1}{\tau }\left( {C^{\prime}_{\varepsilon 1} \left( {\zeta ,\alpha } \right) - C^{\prime}_{\varepsilon 2} \left( {k,\varepsilon ,\alpha } \right)\rho \varepsilon } \right) $$

31 $$ \rho \left( {u.\nabla } \right)\zeta = \nabla .\left[ {\left( {\mu + \frac{{\mu_{T} }}{{\sigma_{\zeta } }}} \right)\nabla \zeta } \right] + \frac{2}{k}\left( {\alpha^{3} \mu + \frac{{\mu_{T} }}{{\sigma_{k} }}} \right)\nabla k.\nabla \zeta + \left( {1 - \alpha^{3} } \right)f_{w} + \alpha^{3} f_{h} - \frac{\zeta }{k}P_{k} $$

where *k, ε* and *ζ* show the turbulent kinetic energy, turbulence dissipation rate, and turbulent relative fluctuations, respectively. The turbulent viscosity (*µ*_*T*_) and production term (*P*_*k*_) were calculated by Eqs. (32) and (33), respectively:

32 $$ \mu_{T} = \rho C_{\mu } k\zeta \tau , \, \tau = \max \left[ {\frac{k}{\varepsilon },C_{\tau } \sqrt {\frac{v}{\varepsilon }} } \right] $$

33 $$ P_{k} = \mu_{T} \left[ {\nabla u:\left( {\nabla u + \left( {\nabla u} \right)^{T} } \right) - \frac{2}{3}\left( {\nabla .u} \right)^{2} } \right] - \frac{2}{3}\rho k\nabla .u $$

The elliptic blending function (*α*) and Reciprocal wall distance (*G*) were obtained by Eqs. (34) and (35), respectively:

34 $$ \alpha - L^{2} \nabla^{2} \alpha = 1, \, L = C_{L} \max \left[ {\frac{{k^{1.5} }}{\varepsilon },C_{\eta } \left( {\frac{{v^{3} }}{\varepsilon }} \right)^{0.25} } \right] $$

35 $$ \nabla G.\nabla G. + \sigma_{w} G\left( {\nabla .\nabla G} \right) = \left( {1 + 2\sigma_{w} } \right)G^{4} , \, l_{W} = \frac{1}{G} - \frac{{l_{ref} }}{2} $$

36 $$ f_{w} = - \zeta \frac{\varepsilon }{k}, \, f_{h} = - \frac{1}{\tau }\left( {C_{1} - 1 + C_{2} \frac{{p_{k} }}{\rho \varepsilon }} \right)\left( {\zeta - \frac{2}{3}} \right) $$

where *C*_1_, *C*_2_, *C*_μ_, *C*_τ_, *C*_η_, $C^{\prime}_{\varepsilon 1}$, $C^{\prime}_{\varepsilon 2}$, *σ*_k_, σ_*w*_, *σ*_ε_ and *σ*_ζ_ are the constant in the above equations. To describe the temperature variation through the 3S, the V2-f turbulent model along with the energy equation were simultaneously solved. Besides, to consider temperature variation, additional terms including viscous dissipation (*ϕ*) and pressure work (*Q*_*p*_) were added in the energy equation. The pressure work (*Q*_*p*_) and viscous dissipation (*ϕ*) were determined by the Eqs. (37) and (38), respectively^46^:

37 $$ Q_{P} = \alpha_{P} Tu.\nabla P_{A} $$

38 $$ \phi = 2\mu \left[ {\left( {\frac{\partial u}{{\partial x}}} \right)^{2} + \left( {\frac{\partial v}{{\partial y}}} \right)^{2} + \left( {\frac{\partial w}{{\partial z}}} \right)^{2} + \frac{1}{2}\left( {\frac{\partial v}{{\partial x}} + \frac{\partial u}{{\partial y}}} \right)^{2} + \frac{1}{2}\left( {\frac{\partial w}{{\partial y}} + \frac{\partial v}{{\partial z}}} \right)^{2} + \frac{1}{2}\left( {\frac{\partial u}{{\partial z}} + \frac{\partial w}{{\partial x}}} \right)^{2} } \right] $$

where:

39 $$ \alpha_{P} = - \frac{1}{\rho }\left( {\frac{\partial \rho }{{\partial T}}} \right)_{P} $$

## Results and discussion

To promote the 3S’s application in the oil and gas industry, it is crucial to optimize its geometry design and operational condition. The developed model provided a clear view of the distribution of temperature, pressure, velocity, and liquid droplets inside the nozzle. In this study, the influence of nozzle structure and operational conditions on the separation performance of 3S were investigated. In the optimal design, the operating conditions can be adjusted in such a way to maximize the cooling performance and collection efficiency. Several geometrical parameters, including the structure of convergence part, diffuser, drainage port, and swirler, were optimized to improve the collection efficiency and decrease the energy loss for a specified operational condition. Table 4 summarizes the optimizations performed in each step.

**Table 4:** Summary of optimizations performed in different sections.

Device	Section	Core diameter	Core length	Vane height	Vane thickness	Number of vanes	Swirl angle	Objective function	Optimized value
Axial swirler	3.2.1	5 cm	10 cm	1.75 cm	0.2 cm	8	10–60°	Swirl angle	40°
	3.2.2	5 cm	10 cm	1.75 cm	0.2 cm	4–16	40	Number of vanes	12
	3.2.3	5 cm	10 cm	0.584–1.75 cm	0.2 cm	12	40	Vane height	1.75 cm
	3.2.4	5 cm	10 cm	1.75 cm	0.1–0.4 cm	12	40	Vane thickness	0.1 cm

Drainage port	Section	Clearance length	Clearance depth	Objective function	Optimized value				
	3.2.5	4 mm	0 – 4 mm	Clearance depth	2 mm				
	3.2.5	2 – 6 mm	2 mm	Clearance length	2 mm				

Item	Section	PRR	Slip gas velocity	Droplet diameter	Inlet temperature	Objective function	Optimized value		
Operating parameter	3.4.1	0.7–0.8	6 m/s	2 µm	278.15 K	PRR	0.766		
	3.4.2	0.766	1–6 m/s	2 µm	278.15 K	Slip gas velocity	6 m/s		
	3.4.3	0.766	6 m/s	0.1–2 µm	278.15 K	Droplet diameter	2 µm		
	3.4.4	0.766	6 m/s	0.1–2 µm	278.15 – 293.15 K	Inlet temperature	283.15 K		

**Figure 5:**
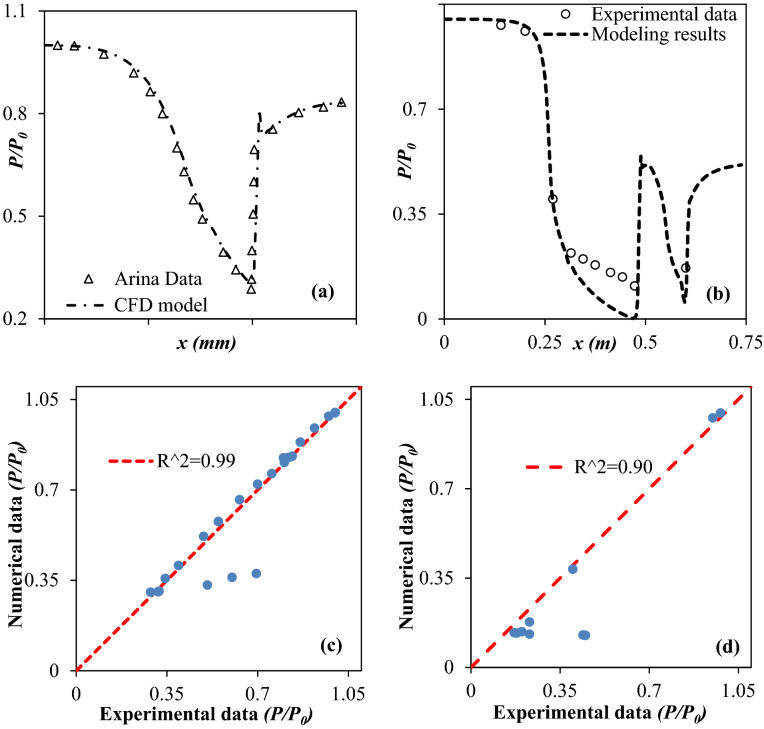
Validation of the developed CFD model: (**a**, **b**) numerical results versus experimental data, (**c**, **d**) error analysis by root-mean-square (R^2^).

Before the developed model was employed to optimize the nozzle structure, it should be validated by suitable experimental data. Two different data sets (^35,47^ in Fig. 5) were used for validation of the developed model. It can be observed that the predicted trend of pressure distribution was consistent with the experimental data. Furthermore, to further investigate the accuracy of the developed CFD model, the root mean square (*R*^2^) was used, which is defined by Eq. (40)^48,49^. Figure 5 shows the results of comparison between numerical results and experimental data. As can be seen in Fig. 5, there is a good agreement between the numerical results and the experimental data, especially for Arina’s data. Therefore, the developed CFD model can suitably predict the supersonic separation behavior through the converging–diverging nozzle.

40 $$ R^{2} = 1 - \frac{{\sum\nolimits_{i = 1}^{n} {\left( {a_{i} - d_{i} } \right)^{2} } }}{{\sum\nolimits_{i = 1}^{n} {\left( {d_{i} } \right)^{2} } }} $$

where *a*_*i*_ is the experimental data, *d*_*i*_ is the numerical result and n is the number of investigated data.

### Optimization of the nozzle structure

The structure of the converging—diverging nozzle was optimized to maximize the cooling performance and minimize the pressure loss across the nozzle. The optimized structure increased the separation efficiency by improving the cooling performance for a specified operating condition. Furthermore, with the decrease of the average temperature through the convergent section, the domain of the low-temperature region extended and resulted in the increase of liquid droplets diameter. Therefore, the optimization of the nozzle structure was conducted based on two factors: (a) Minimization of average cooling temperature and (b) decreasing the minimum achievable temperature. The minimum temperature characterized the expansion properties of the 3S. The average cooling temperature (through the Laval nozzle and constant cross-sectional area channel) was calculated by the Eq. (40):

40 $$ \delta = \iiint {T_{x} dV} $$

**Figure 6:**
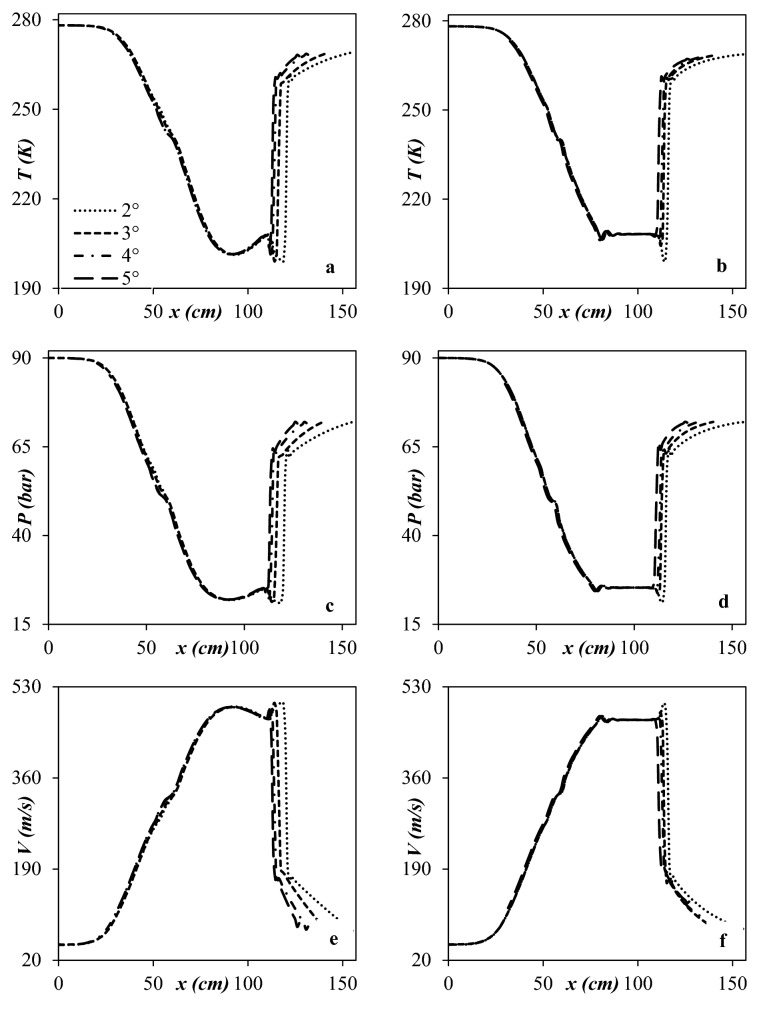
Temperature (**a**, **b**), pressure (**c**, **d**) and velocity (**e**, **f**) profile for two different line types (**a**, **c**, **e**) Curved wall diffuser (**b**, **d**, **f**) Linear wall diffuser.

As a first step, several cases were studied for a specified operational condition to optimize the nozzle structure. It is evident that the structure of the convergent section and diffuser has a significant influence on the temperature profile and flow field inside the 3S. The role of the diffuser is converting the kinetic energy to pressure energy. The influence of the divergent angle of the diffuser was also investigated in this study. To optimize the diffuser structure, a fixed geometry was employed for the Laval nozzle. During optimization of diffuser structure, Bi-cubic’s analytical formula was used to design the convergent part of the 3S. Also, two different line-types, including curved wall and linear wall, were considered for the diffuser section. The temperature, pressure, and velocity distribution from inlet to outlet are presented in Fig. 6. It can be found that the shockwave position was strongly influenced by the diffuser angles. The simulation results showed that with an increase of divergent angle, the shockwave position moves backward. For a fixed outlet diameter of the nozzle, the divergent angle of the diffuser characterized the domain of the low-temperature region. The cooling performance, liquid droplets size, and the amount of the liquid phase inside the nozzle are influenced by the divergent angle. Furthermore, as shown in Fig. 6, as the diffuser angle increases, the minimum temperature and the average cooling temperature at the diffuser rise, which resulted in deterioration of cooling performance. In addition, as the diffuser angle decreases, the velocity distribution becomes more uniform. Therefore, increasing or decreasing the diffuser angle has significant influences on the velocity, pressure, and temperature distribution through the nozzle.

In conclusion, simulation results demonstrated that the diffuser with divergent angle of 2° is a good choice. To improve the uniformity of natural gas flow, the variation of the profile curve in the diffuser section should be smooth. Figure 6 presents the axial distribution of velocity, pressure, and temperature for curved wall and linear diffuser, respectively. Figure 6 illustrates that for the curved wall diffuser, the temperature and pressure variation was smoother than the linear wall diffuser. Furthermore, for the divergence angle of 2°, the shockwave location for the linear wall diffuser was ahead of the curved wall diffuser, which means that the domain of the low-temperature region was larger for the curved wall diffuser. In conclusion, the obtained minimum temperature was lower and then the cooling performance was higher for the curved wall diffuser than the linear wall diffuser (Fig. S.2). Therefore, the optimization results illustrated that the curved wall diffuser with the divergent angle of 2° is the best choice for improving the cooling performance and flow stability.

**Figure 7:**
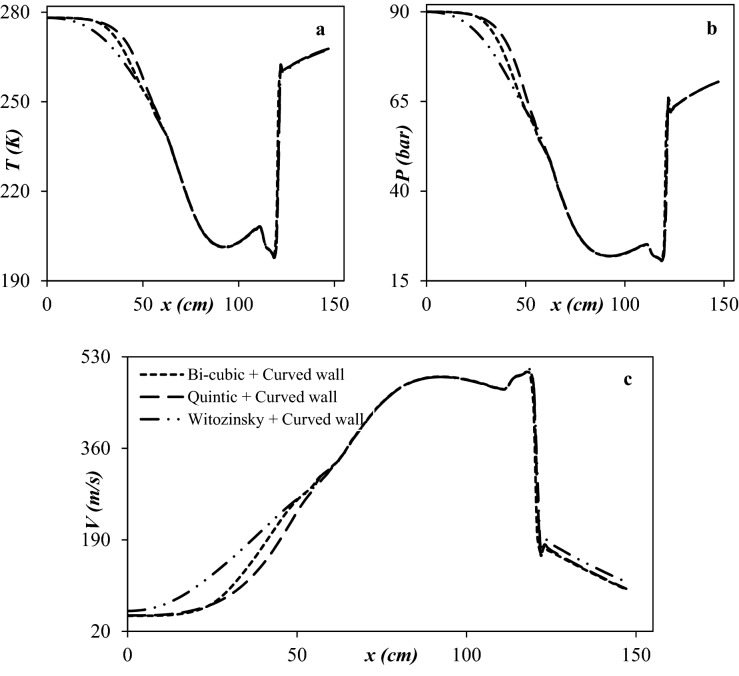
Temperature (**a**), pressure (**b**) and velocity (**c**) profile for three different line types of convergent section.

The main role of the Laval nozzle is decreasing the natural gas temperature to a suitable level that is appropriate for the condensation of water vapor and heavy HCs. The cooling performance of the Laval nozzle depended on the line type design of the convergent part. Therefore, it is crucial to optimize the structure of the convergent section. In this study, the Bi-cubic curve, Quintic curve, and Witoszynski curve were employed to design the line-type of convergent section. Simulation results demonstrated that the Witoszynski curve exhibited the lowest average cooling temperature compared to the Bi-cubic curve and Quintic curve. It could be observed (Fig. 7) that the average cooling temperature by Witoszynski curve was about 242.1 K, while these values were about 242.9 K and 243.8 K for Bi-cubic and Quintic convergent curve, respectively. Moreover, Fig. 7a demonstrates that the length of the low temperature through the convergent section was longer for the Witoszynski curve compared to the other profile curve. Therefore, the Witoszynski curve and the curved wall diffuser (with 2° divergent angle) were employed to design the convergent section and diffuser, respectively. For this optimal nozzle the minimum temperature and maximum velocity were about 197.85 K and 506.2 m/s, respectively.

### Optimization of the swirler structure

**Figure 8:**
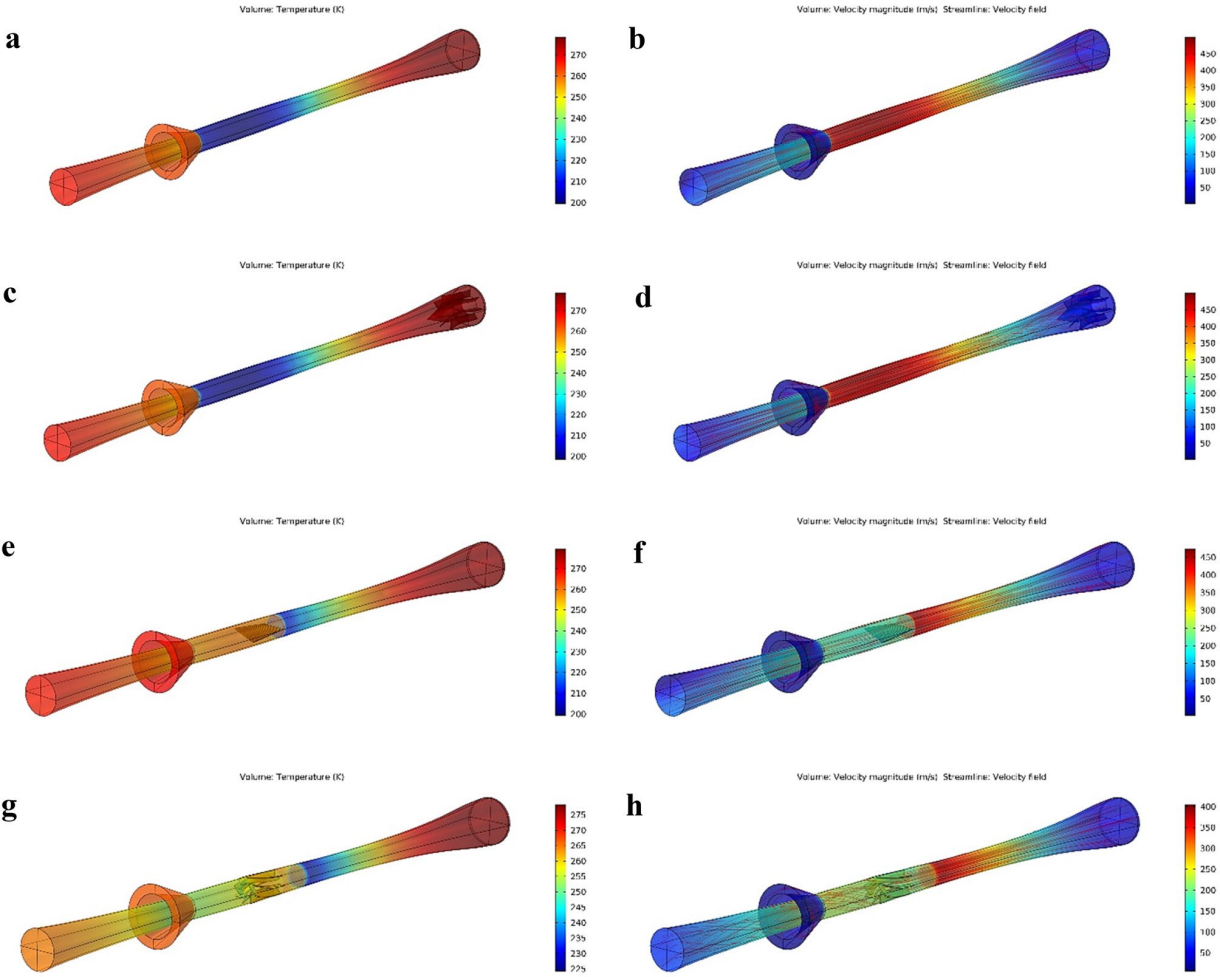
Temperature (**a**, **c**, **e**, **g**) and velocity (**b**, **d**, **f**, **h**) profiles for simple 3S (a, **b**), 3S quipped with axial swirler (**c**, **d**), 3S quipped with helical swirler (**e**, **f**) and 3S quipped with wall mounted swirler (**g**, **h**).

The effect of swirler geometrical parameters such as swirling angle, number of the static vanes, the height of static vane on the temperature profile, and shockwave position were investigated to provide the highest separation efficiency. Three different structures, including axial, wall-mounted, and helical, were considered for the swirler in this section. The collection efficiency was only about 12% for the simple nozzle (3S without swirler). According to the results, the collection efficiency was significantly improved when the simple nozzle was equipped with the swirler. In addition, Fig. 8 demonstrates that the refrigeration temperature was lower and the maximum velocity was higher for the simple nozzle compared to the nozzle equipped with a swirler. In conclusion, installing the swirler improved the collection efficiency while weakening the cooling performance of the 3S. This phenomenon is due to the fact that part of the natural gas energy was consumed to create radial rotation instead of being spent on cooling. Therefore, the swirl intensity and cooling performance are mutually exclusive. Consequently, the separation efficiency (γ) was defined to find the balance point between the cooling performance and collection efficiency.

**Figure 9:**
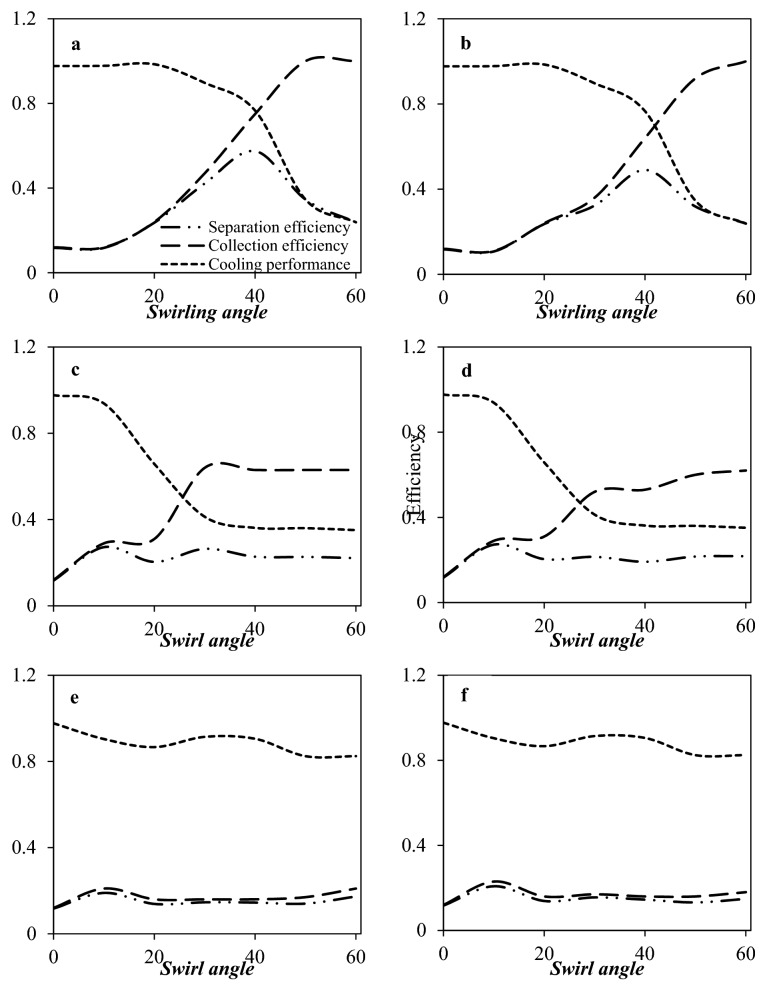
The influence of swirl angle on the collection efficiency, cooling performance and separation efficiency of 3S quipped with axial swirler (**a**, **b**), wall mounted swirler (**c**, **d**) and helical swirler (**e**, **f**) for water (**a**, **c**, **e**) and HC droplets (**b**, **d**, **f**).

As shown in Figs. 8 and 9, the type and location of the swirler have significant effects on the temperature profile and separation efficiency. Simulation results demonstrated that the separation efficiency was higher for the axial swirler compared to the wall-mounted and helical swirler. In conclusion, the swirl intensity was significantly higher for the axial swirler than the wall-mounted and helical swirler. However, for helical and wall-mounted swirler the centrifugation occurred, but it was minimal to direct the liquid droplets toward the separator wall. Therefore, most of the liquid droplets were escaped from the dry gas outlet. Consequently, the axial swirler better generated the centrifugal force compared to the helical and wall-mounted swirler (under similar operational conditions). Installing helical and wall mounted swirler destroyed the low-temperature region and influenced on the condensation characteristics of the 3S. The main advantage of axial swirler was its location of the generation of swirling flow. For the axial swirler, the swirling flow was generated at the entrance, while for the helical and wall-mounted swirler, the swirling flow were generated at the middle of the nozzle where the flow turbulency is very high. Another advantage of axial swirler was the nozzle cross-section varied from circular shape to annular shape. In conclusion, the swirl strength improved considerably for the axial swirler. For the axial swirler, both the cooling performance and separation efficiency were reasonable. At the swirling angle of 50°, the collection efficiency becomes 100% for the axial swirler. Therefore, Due to the large energy loss of helical and wall-mounted swirler and higher separation efficiency of axial swirler, the axial swirler was considered for the next discussions.

#### Optimization of swirling angle

In this section, the CFD modeling was used to optimize the exit angle of the axial swirler. Figure 9 shows the collection efficiency and cooling performance under the condition that the swirl angle was 10°, 20°, 30°, 40°, 50°, and 60°, respectively. There is an optimum value for the swirl angle. In this angle, the best separation efficiency is achieved. It can be observed that when the swirling angle was low, the induced centrifugal force was not enough to direct the liquid droplets toward the separator wall. Therefore, they were escaped from the dry gas outlet. Simulation results showed that by increasing the swirl angle, the collection efficiency improved significantly. Simultaneously, the minimum temperature increased, which means that the cooling performance was deteriorated due to the swirling motion. For example, the minimum temperature was about 259.01 K, when the swirl angle was 60°, while it reduced to 199.64 K when the swirl angle was about 10°. Therefore, it is necessary to found the balance point between the cooling performance and the collection efficiency. Figure 9a and b illustrate that by increasing the swirl angle, the separation efficiency enhanced initially and reached a peak value and then decreased. Therefore, this peak value (swirl angle of 40°) was selected as the optimum angle. In conclusion, the swirl angle of 40° was recommended for the generation of swirling flow inside the 3S. This conclusion was obtained based on the comprehensive analysis of cooling performance and collection efficiency.

**Figure 10:**
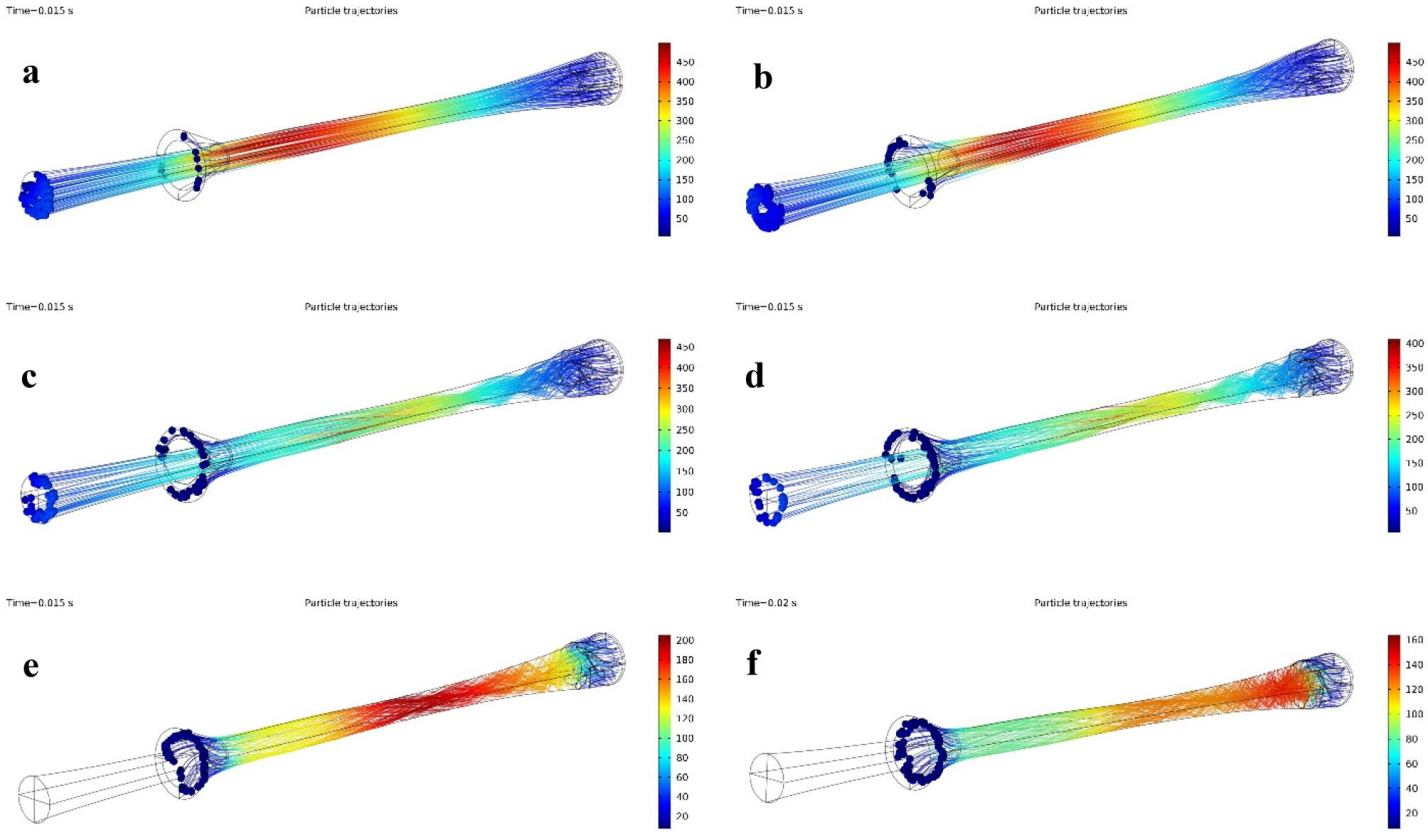
Droplet trajectories and velocity profile for various swirl angles (**a**) 10°, (**b**) 20°, (**c**) 30°, (**d**) 40°, (**e**) 50°, (**f**) 60°.

Figure 10 shows that when other operational and structural parameters remained constant with increasing the swirl angle, the shockwave position shifted toward the nozzle entrance. Moreover, it can be observed that at a high swirl angle (swirl angle of 60°) no obvious shockwave has been appeared inside the nozzle. This is due to the generation of the great obstacles to the natural gas flow. Simultaneously, simulation results demonstrated that as the swirl angle increased, the non-uniformity in the velocity profile became more apparent.

**Figure 11:**
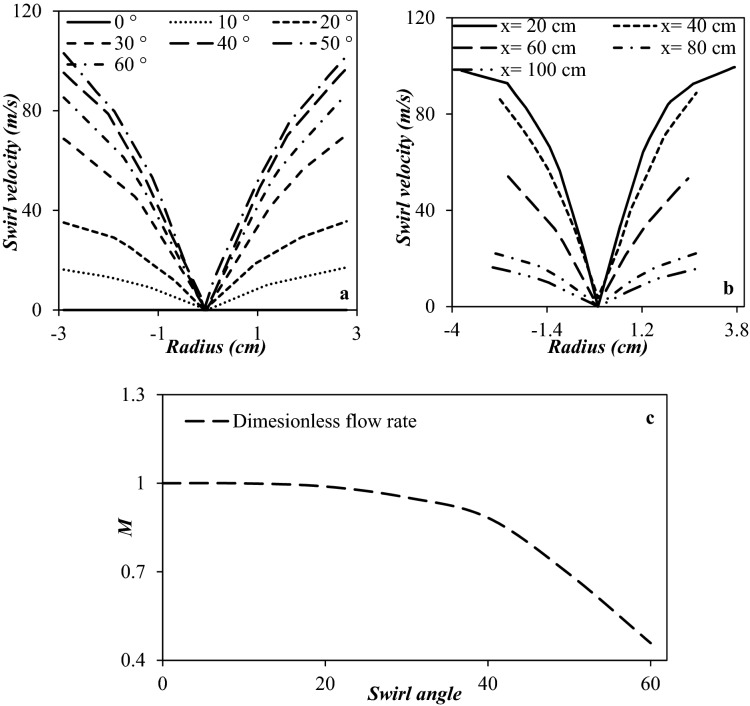
Swirl velocity (at x = 0.35 m) (**a**, **b**) and dimensionless mass flow (**c**) rate for (**a**, **c**) various swirl angles (**b**) swirl angle of 40° at various locations.

The swirl angle is an important factor influencing on the centrifugal force strength. Figure 11a and b depict the swirl velocity for different swirl angles (Fig. 11a) and locations (Fig. 11b), respectively. It can be observed that the swirl angle greatly influenced on the swirl velocity and centrifugal force. Simulation results showed that by increasing the swirl angle, the strength of swirl flow improved up to a specified point. For example, the maximum swirl velocity was about 17 m/s and 96.9 m/s for swirl angle of 10° and 40°, respectively (at x = 0.35). This value decreased to 86.6 m/s for the swirl angle of 60°. As can be seen in Fig. 11, after reaching the swirl angle of 50°, with increasing the swirl angle, the swirl velocity decreased. In another word, at the swirl angles of 50°, the highest swirl velocity at the separator wall was observed. This issue occurs because as the swirl angle increases, the rate of energy loss increases dramatically. Furthermore, as shown in Fig. 11b for a specified swirl angle, the swirl velocity decreased along the central axis of the nozzle. This phenomenon occurred due to the friction of the natural gas with the separator wall.

A dimensionless parameter (M) was defined to study the influence of variation of swirl angle on the mass flow rate as follow:

47 $$ M = {\raise0.7ex\hbox{${m_{S} }$} \!\mathord{\left/ {\vphantom {{m_{S} } {m_{0} }}}\right.\kern-\nulldelimiterspace} \!\lower0.7ex\hbox{${m_{0} }$}} $$

where *m*_*0*_ and *m*_*S*_ are the mass flow rate of a simple 3S and a 3S equipped with the swirler, respectively. Figure 11c shows the dimensionless mass flow rate versus the swirl angle. The deviation of M from unity shows the influence of the swirl strength on the mass flow rate. It is evident that the mass flow rate reduced with increasing swirl intensity. This reduction in mass flow rate occurred due to the high swirl angle generated a large energy loss for natural gas flow through the nozzle.

#### Optimization of the number of static vane cone swirler structure

**Figure 12:**
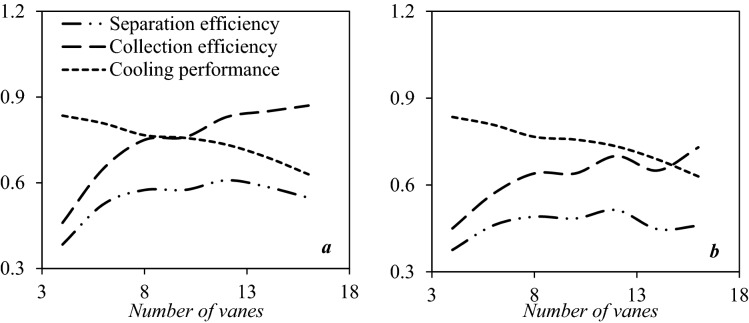
The influence of number of static vanes on the collection efficiency, cooling performance and separation efficiency of 3S for (**a**) water droplets and (**b**) HC droplets.

In this section, the influence of the number of installed static vanes on the swirl strength and separation efficiency were investigated numerically. The number of static vanes plays a crucial role in the generation of swirling flow. Figure 12 shows the distribution of cooling performance, collection efficiency, and separation efficiency when the number of static vanes was 4, 6, 8, 10, 12, 14, and 16, respectively. It can be seen that the collection efficiency was improved, and the cooling performance deteriorated as the number of static vanes was increased. Besides, the separation efficiency firstly improved and then deteriorated as the number of the static vanes increased. Generally, due to the high swirl intensity, the energy loss in the nozzle increased, which reduced the cooling performance and condensation area through the nozzle. This issue is disadvantageous for the formation of liquid droplets. Therefore, under the influence of the moderate swirl intensity, suitable expansion characteristics along with high centrifugal force were provided in the 3S. Simulation results demonstrated that when the number of static vanes reached 12, the highest separation efficiency was obtained. Therefore, 12 static vanes were considered for the rest of the paper. Furthermore, under constant operational condition (PRR = 0.8), when the number of static vanes shifted from 4 to 16, the location of shockwave varied from x = 0.589 m to x = 0.47 m, which means that due to the energy loss, the shockwave position back stepped to the nozzle entrance and the domain of low-temperature region narrowed.

The natural gas swirl velocity (versus nozzle radius) at the downstream of the static vanes (at x = 0.35 m) were presented in Fig. S.3a. Generally, when the number of static vanes increased, the swirling velocity enhanced significantly. It should be stated that there is a direct relationship between the maximum value of centrifugal acceleration and swirl number^31^. Fig. S.3b shows the variation of swirl velocity at the different axial locations inside the nozzle. It can be seen that due to the friction between the gas phase and separator wall, the swirl strength was weakening along the length of the nozzle. Therefore, in the designing of 3S, a reasonable length should be employed to achieve a balance condition between the residence time (for the formation of liquid droplets) and reduction of centrifugal acceleration along the nozzle length. Meanwhile, the swirl velocity increased from the center of the nozzle to the separator wall, which is suitable for directing the liquid droplets toward the separator wall. In addition, the influence of the number of static vanes on the mass flow rate was studied. Simulation results showed that the mass flow rate decreased with the rise of the number of static vanes.

#### Optimization of vane height

**Figure 13:**
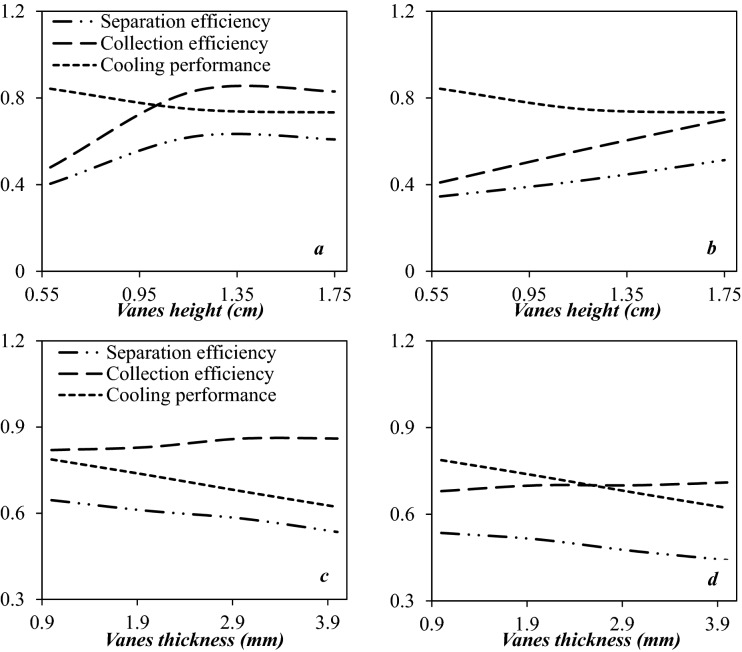
The influence of static vanes height (**a**, **b**) and static vanes thickness (**c**, **d**) on the collection efficiency, cooling performance and separation efficiency of 3S for (**a**, **c**) water droplets and (**b**, **d**) HC droplets.

The height of the static vane was varied to analyze its influence on the flow field and separation efficiency by selecting three different heights (h = 0.584, 1.167, and 1.75 cm). Figure 13a and b shows that the influence of static vanes height on the cooling performance, collection efficiency, and separation efficiency. Generally, the effect of increasing the vane height on the separation efficiency was negligible compared to other investigated parameters. As shown in Fig. 13a, b, by increasing the height of static vanes the separation efficiency improved for HC droplets. Contrary to this, for water droplets, the separation efficiency slightly changed when the height of static vanes increased from 1.167 to 1.75 cm. Therefore, decreasing the static vane height resulted in the droplets did not direct by the static vane and moved straight forward along the axial direction. In conclusion, the optimal static vane height of 1.75 cm was selected for the rest of the paper. It should be emphasized that while enhancing the vane height improved the collection efficiency, it is important to consider that this modification may lead to deterioration of cooling performance due to the high energy loss.

#### Optimization of vane thickness

The static vane thickness was the next structural parameter whose effect on separation efficiency was examined. Figure 13c and d shows the effect of static vane thickness on the separation efficiency. In this case, like the height of the static vane, the thickness of the static vane has little effect on the separation efficiency. With the decrease of the static vane thickness, the minimum temperature gradually decreased, which resulted in improving liquefaction rate and separation efficiency. The reason is that when the vane thickness is decreased, its influence on the flow field is reduced. In addition, the separation efficiency slightly improved by decreasing the static vane thickness. Considering these criteria, the vane thickness of 1 mm was selected for the rest of the paper.

#### Optimization of drainage port structure

**Figure 14:**
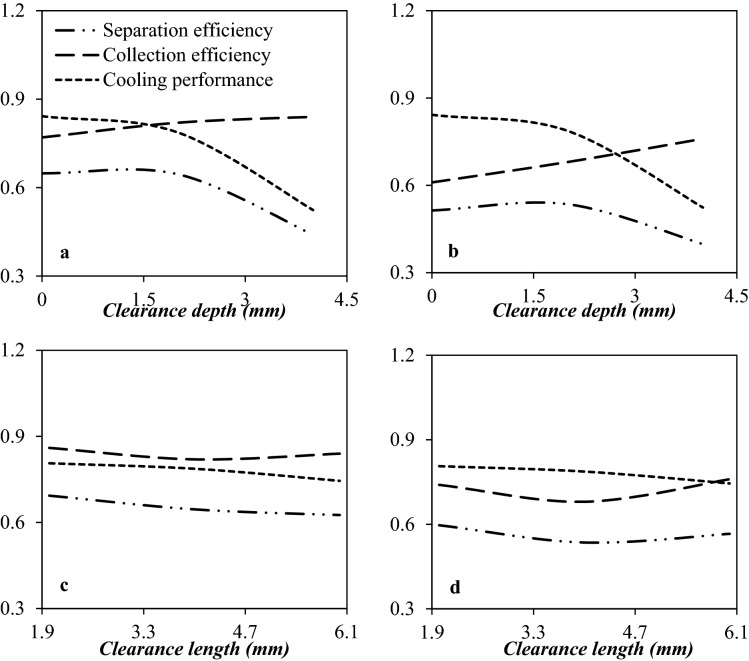
The influence of clearance depth (**a**, **b**) and clearance length (**c**, **d**) on the collection efficiency, cooling performance and separation efficiency of 3S for (**a**, **c**) water droplets and (**b**, **d**) HC droplets.

After the liquid droplets are accumulated in the separator wall, they are coalesced into each other and formed a liquid film. These droplets are separated from the gas phase at the drainage port. The structure of the drainage port has significant influences on the flow field in the divergent section of the nozzle. It is evident, different clearance depths have different influences on the nozzle performance. There is an optimal clearance depth that is more appropriate for liquefaction and separation of the liquid droplets. Simulation results demonstrated that the non-uniformity of gas flow along the radial direction was higher for the internal extension structure (clearance depth ˃ 0 mm) compared to the flush type structure (clearance depth = 0 mm). Also, the internal extension structure had a significant effect on the flow field and weakened the shockwave, and destroyed the cooling performance. The reason for this issue was the increase of turbulence in the flow of natural gas through the 3S. The effect of the drainage port type on the separation efficiency was shown in Fig. 14. It can be observed that the 3S equipped with an internal extension structure showed higher collection efficiency than the 3S equipped with a flush-type drainage structure. In addition, the expansion characteristic of the separator equipped with a flush type drainage structure was better than the separator equipped with an internal extension drainage structure. Simulation results demonstrated that increasing the clearance depth improved the collection efficiency, while the cooling performance deteriorated. Generally, it is crucial to explore the balance point between the cooling performance and the collection efficiency. As shown in Fig. 14, the effect of collection efficiency was greater than that of cooling performance up to a clearance depth of 2 mm (*L*_*depth*_ = 2 mm). Consequently, the separation efficiency in the nozzle equipped with an internal extension structure was slightly higher than the nozzle equipped with a flush-type drainage structure up to a clearance depth of 2 mm. In conclusion, while the flush type drainage structure has a negligible effect on the flow field and velocity profile inside the nozzle, but the separation efficiency was lower for this structure. This conclusion was observed in previously published work^50^. Therefore, the internal extension structure with a clearance depth of 2 mm, showed higher separation efficiency than the flush type structure for the dehydration and NGL recovery by the 3S.

Another parameter that was examined in this section was the clearance length. The investigated clearance length was 2 mm, 4 mm, and 6 mm, respectively. The influence of the clearance length size on the separation efficiency was presented in Fig. 14. It was observed that the separation efficiency was influenced by the clearance length of the drainage port. As the gap size increases, more liquid droplets can directly enter the drainage port and be separated from the continuous phase. Therefore, the collection efficiency was improved for larger gap sizes, whereas the cooling performance deteriorated. This decrease in cooling performance occurred due to the disturbance of the flow field inside the nozzle. It is evident that the effect of both parameters should be considered simultaneously. Simulation results demonstrated that as the gap size increases from 2 to 6 mm, the nozzle separation efficiency decreases from (a) 59.6% to 56.6% for HC droplets and (b) 69.3% to 62.5% for water droplets. As a result, the influence of cooling performance was more significant, and increasing the clearance length caused a decrease in separation efficiency, and it was not desirable. Therefore, through a complete examination of cooling performance and collection efficiency, we suggested the clearance length of 2 mm for the drainage port.

### The influence of inserting inner body

In this section, the influence of installing the central body on the separation efficiency was investigated. The central body (central core) was inserted inside the nozzle to preserve the principle of conservation of angular momentum. It was observed that installing the central body had a significant influence on the collection efficiency of the 3S. This conclusion was consistent with previously published work^12^. Simulation results demonstrated that the central body significantly improved the separation efficiency of the nozzle from (a) 69.3% to 81.4% for water droplets and (b) 59.6% to 65.1% for HC droplets. This improvement in separation efficiency resulted from an improvement in the collection efficiency. In addition, the cooling performance was slightly improved.

### The influence of operational parameters

In the previous section, the structural parameters were optimized. The so far geometrically optimized structure was employed to optimize the operating condition through the nozzle. For industrial applications, decreasing the pressure loss for a specified cooling target is very important. Therefore, the combination of collection efficiency and cooling performance were considered in this section to optimize the operational parameters through the nozzle correctly.

#### The influence of PRR on the separation efficiency

In this section, the inlet temperature and pressure remained constant while the outlet pressure varied from 63 to 72 bar. Four different scenarios were defined for the PRR (PRR = 0.7, 0.733, 0.766, and 0.8). In order to consider the effect of PRR, a new definition which is called “total separation efficiency” was defined as follows:

48 $$ Total \, separation \, efficiency\left( \% \right) \, = \, separation \, efficiency\left( \% \right) \times \left( {{{PRR} \mathord{\left/ {\vphantom {{PRR} {0.8}}} \right. \kern-\nulldelimiterspace} {0.8}}} \right) $$

**Figure 15:**
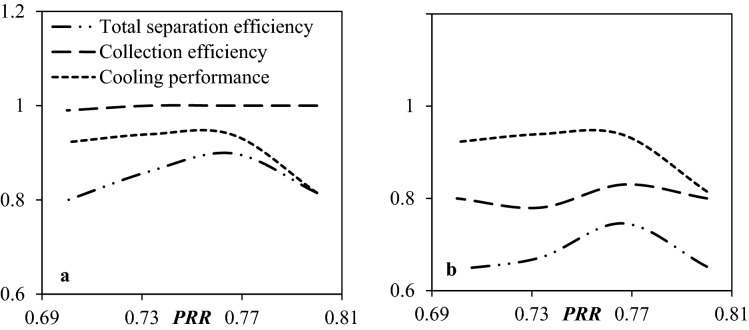
The influence of PRR on the collection efficiency, cooling performance and total separation efficiency of 3S for (**a**) water droplets and (**b**) HC droplets.

It can be observed that decreasing the PRR from 0.8 to 0.766 improved both the cooling performance and the total separation efficiency (Fig. 15). Figure 15 illustrates that when the PRR became less than 0.8, the total separation efficiency increased and then gradually decreased with this variation. It can be found that the highest total separation efficiency of water droplets can reach about 89.8% for the PRR = 0.766. As the PRR decreases, the expansion characteristics through the nozzle improve. This reduction in PRR not only guaranteed a better operating condition for liquefaction of the condensable components but also the length of the low-temperature region increased significantly. Furthermore, simulation results demonstrated that with the variation of the PRR, the swirl velocity changed slightly.

**Figure 16:**
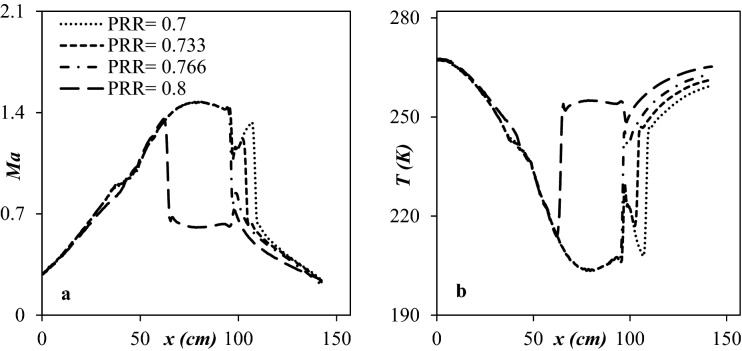
The influence of PRR on the shockwave position for (**a**) Ma number and (**b**) Temperature.

Figure 16 shows the temperature profile and Ma number through the nozzle’s length for various outlet pressures. One of the main factors that influence the separation efficiency is the shockwave position^51^, and this position is a function of PRR. Figure 16 illustrated that the shockwave position moved toward the outlet as the PRR decreased. After the shockwave incidence, due to the enhancement of operating temperature, the condensed liquid droplets will be re-evaporated into the gas phase. Consequently, the PRR should be varied to adjust the shockwave position close to the drainage port correctly. In the improved structure, when the PRR was decreased to 0.766, the shock was located at x = 110.2 cm, which was close to the drainage port. Therefore, the PRR of the optimized structure should be about 0.766 to operate appropriately.

#### The influence of slip gas flow rate on the separation performance

**Figure 17:**
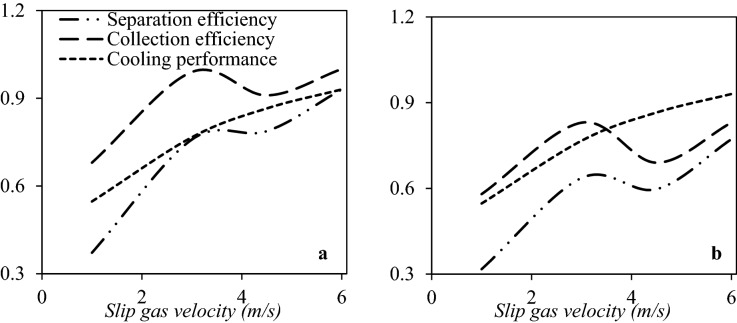
The influence of slip gas velocity on the collection efficiency, cooling performance and separation efficiency of 3S for (**a**) water droplets and (**b**) HC droplets—(PRR = 0.766).

**Figure 18:**
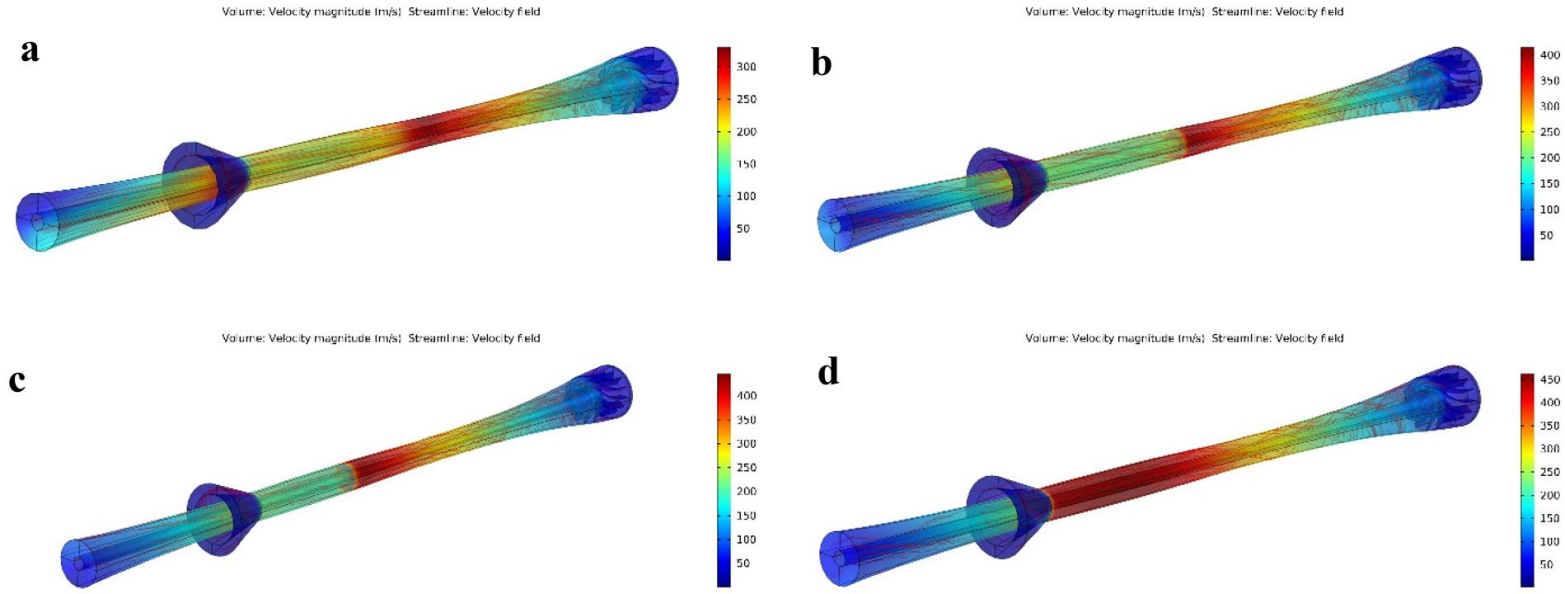
The influence of slip gas velocity on the shockwave position (**a**) V = 1 m/s, (**b**) V = 3 m/s, (**c**) V = 4.5 m/s, (**d**) V = 6 m/s—(PRR = 0.766).

In the drainage port, there is some slip gas with the liquid phase. Figure 17 presents the effect of slip gas velocity on the cooling performance, collection efficiency, and separation efficiency (for PRR = 0.766). It was observed that the slip gas flow rate affected the temperature, pressure, and velocity profile inside the nozzle. Therefore, in addition to PRR, the amount of slip gas flow rate also influenced the shockwave position. When the slip gas velocity reached 4.5 m/s, the collection efficiency was damaged due to the degradation of the swirl velocity (Fig. S.4). In addition, as shown in Fig. 18, an increase in the slip gas velocity shifted the shockwave position toward the separator outlet, and then the cooling performance improved. This improvement occurred due to the displacement of the shockwave position and then enhancing the low-temperature region. This shock wave position displacement was due to the extraction of some of the working fluid at the drainage port. This extraction resulted in the decrease of the energy loss of the working fluid due to the reduction in the volumetric flow rate of natural gas passing through the nozzle. In conclusion, the overall friction was reduced. Therefore, as shown in Fig. 17, with increasing slip gas velocity and reaching 6 m/s, the highest separation efficiency was obtained, and this velocity (6 m/s) was selected as the optimal value.

#### The influence of droplet size and density on the separation efficiency

**Figure 19:**
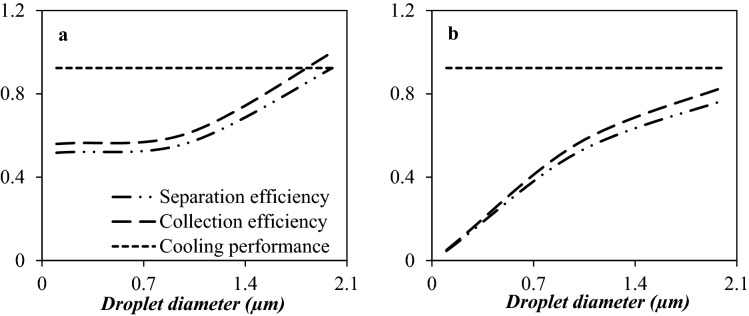
The influence of droplet size and density on the collection efficiency, cooling performance and separation efficiency of 3S for (**a**) water droplets and (**b**) HC droplets—(PRR = 0.766).

As the density ratio of the liquid phase to the gas phase increases, the strength of centrifugal forces enhances, and then the separation efficiency improves. Figure 19 presents the influence of droplet size and density on the separation efficiency for droplets with diameter ranges from 0.1 to 2 µm. The water droplets were located closer to the separator wall (than HC droplets) due to the induced centrifugal force. Contrary to this, the HC droplets were located closer to the nozzle central axis and escaped with the dry gas. In conclusion, the induced centrifugal force augmented with density. In addition, as shown in Fig. 19, the liquid droplet size played a crucial role in the separation performance. The liquid droplet size influences the induced centrifugal force^52^. Based on the Newton’s law of mechanics, the induced centrifugal force on a larger droplet is higher than a smaller one. Figure 19 demonstrates that with the increase of the liquid droplet diameter, the separation efficiency significantly improved. This improvement in separation efficiency occurred due to the improvement in the collection efficiency. The tiny droplets (0.1 µm) were easily entrained by the continuous phase toward the separator outlet, which resulted in the deterioration of separation performance. For liquid droplets with a medium size (1 µm), a small portion of them was carried by the gas phase toward the outlet, while for larger droplets (2 µm), most of them were separated from the gas phase easily. For generation of larger liquid droplets, the low-temperature region inside the Laval nozzle should be enlarged.

#### The influence of inlet temperature on the separation efficiency

To analyze the influence of inlet temperature on the nozzle performance, the separation efficiency under different inlet temperatures (*T* = 278.15, 283.15, 288.15, and 293.15 K) were plotted in Fig. S.5. It can be observed that by increasing the inlet temperature, the separation efficiency increased initially and reached a peak value (*T* = 283.15 K) and then decreased. The cooling performance showed the same behavior as the separation efficiency. Furthermore, it can be observed that the influence of inlet temperature on the collection efficiency was negligible for a constant PRR (PRR = 0.766). Furthermore, decreasing the inlet temperature resulted in a decline in the minimum temperature. Another important parameter was the location of the shockwave. Simulation result illustrated that with the decrease of the inlet temperature, the shockwave position moved backward.

## Conclusion

In this study, the accuracy of seven different turbulence models in predicting high swirling turbulent flow in the supersonic separator were compared. It was observed that the V2-f turbulence model was more accurate than other investigated turbulence models. Once the optimum turbulence model and particle tracing model were determined, the effects of operational and structural parameters on the separation efficiency were examined. The simulation results demonstrated that the combined usage of the Witozinsky line-type (in the convergent section) and the curved-wall diffuser (with a divergence angle of 2°) showed the optimum cooling performance compared to other combinations of convergent section line-type and diffusers. On the other hand, the effect of installing the optimal axial swirler on the separation efficiency for the optimum combination of the convergent section line-type and the curved-wall diffuser was also studied. It was found that under the action of the swirler, the cooling performance deteriorated while the collection efficiency improved significantly. Consequently, the separation efficiency was introduced to find the balance point between the cooling performance and collection efficiency. The separation efficiency for both water and hydrocarbon droplets was improved from 12% in the presence of a featureless nozzle to nearly 64% for water droplets and 53.5% for hydrocarbon droplets in the presence of the optimized axial swirler. Furthermore, it was observed that the structure of the drainage port had significant effects on the flow field in the divergent section of the nozzle. Through a complete examination of the cooling performance and collection efficiency, the clearance depth and clearance length of 2 mm each was suggested for the drainage port. In addition, it was observed that increasing the liquid droplet size and density improved the separation efficiency of supersonic separator. However, there was an optimal point for the inlet temperature and pressure recovery ratio beyond which the separation efficiency declined.

## Supplementary Information

Supplementary Information.
