# Supplementary material for: A novel strategy for comprehensive optimization of structural and operational parameters in a supersonic separator using computational fluid dynamics modeling
Source: Sci Rep. 2021 Nov 8;11:21850. doi: 10.1038/s41598-021-01303-5 (PMC8575786; doi:10.1038/s41598-021-01303-5)
Supplement: Supplementary file 1 — Supplementary Information. [file 41598_2021_1303_MOESM1_ESM.docx]

**A novel strategy for comprehensive optimization of structural and operational parameters in a supersonic separator using computational fluid dynamics modeling**

**Sina Nabati Shoghl^a^, Abbas Naderifar^a*^, Fatola Farhadi^b*^, Gholamreza Pazuki^a^**

^a^Department of Chemical Engineering Amirkabir University of Technology (Tehran Polytechnic)Tehran, Iran

^b^Department of Chemical and Petroleum Engineering, Sharif University of Technology, Azadi Ave., Tehran, Iran

**^*^Corresponding authors:** Abbas Naderifar, Fatola Farhadi (email addresses: [Naderifar@aut.ac.ir](mailto:Naderifar@aut.ac.ir); [farhadi@sharif.edu](mailto:farhadi@sharif.edu))

**Fig. S.1**

|  |  |
| --- | --- |
| **Fig. S.1.** The curvature of convergent section (left) and diffuser section (right). | |

**Fig. S.2**

|  |
| --- |
| **Fig. S.2.** The cooling performance at different divergent angles. |

**Fig. S.3**

| **a** | **b** |
| --- | --- |
| **Fig. S.3.** Swirl velocity for a) various number of static vanes at x=0.35 m and b) 12 static vanes at various locations. | |

**Fig. S.4**

|  |
| --- |
| **Fig. S.4.** Swirl velocity for various slip gas velocities |

**Fig. S.5**

| **a** | **b** |
| --- | --- |
| **Fig. S.5.** The influence of inlet temperature on the collection efficiency, cooling performance and separation efficiency of 3S for a) water droplets and b) hydrocarbon droplets (PRR=0.766). | |
